# A non-synonymous SNP within the *isopentenyl transferase* 2 locus is associated with kernel weight in Chinese maize inbreds (*Zea mays* L.)

**DOI:** 10.1186/1471-2229-13-98

**Published:** 2013-07-05

**Authors:** Jianfeng Weng, Bo Li, Changlin Liu, Xiaoyan Yang, Hongwei Wang, Zhuanfang Hao, Mingshun Li, Degui Zhang, Xiaoke Ci, Xinhai Li, Shihuang Zhang

**Affiliations:** 1 Institute of Crop Science, Chinese Academy of Agricultural Sciences, National Key Facility for Crop Gene Resources and Genetic Improvement, Beijing 100081, China; 2 Shenyang Agricultural University, Shenyang 110161, China; 3 Chongqing Academy of Agricultural Sciences, Chongqing 409912, China

**Keywords:** Maize, Isopentenyl transferase 2, Association mapping, Artificial selection

## Abstract

**Background:**

Kernel weight, controlled by quantitative trait loci (QTL), is an important component of grain yield in maize. Cytokinins (CKs) participate in determining grain morphology and final grain yield in crops. *ZmIPT2*, which is expressed mainly in the basal transfer cell layer, endosperm, and embryo during maize kernel development, encodes an isopentenyl transferase (IPT) that is involved in CK biosynthesis.

**Results:**

The coding region of *ZmIPT2* was sequenced across a panel of 175 maize inbred lines that are currently used in Chinese maize breeding programs. Only 16 single nucleotide polymorphisms (SNPs) and seven haplotypes were detected among these inbred lines. Nucleotide diversity (*π*) within the *ZmIPT2* window and coding region were 0.347 and 0.0047, respectively, and they were significantly lower than the mean nucleotide diversity value of 0.372 for maize Chromosome 2 (*P* < 0.01). Association mapping revealed that a single nucleotide change from cytosine (C) to thymine (T) in the *ZmIPT2* coding region, which converted a proline residue into a serine residue, was significantly associated with hundred kernel weight (HKW) in three environments (*P* <0.05), and explained 4.76% of the total phenotypic variation. *In vitro* characterization suggests that the dimethylallyl diphospate (DMAPP) IPT activity of ZmIPT2-T is higher than that of ZmIPT2-C, as the amounts of adenosine triphosphate (ATP), adenosine diphosphate (ADP), and adenosine monophosphate (AMP) consumed by ZmIPT2-T were 5.48-, 2.70-, and 1.87-fold, respectively, greater than those consumed by ZmIPT2-C. The effects of artificial selection on the *ZmIPT2* coding region were evaluated using Tajima’s D tests across six subgroups of Chinese maize germplasm, with the most frequent favorable allele identified in subgroup PB (Partner B).

**Conclusions:**

These results showed that *ZmIPT2*, which is associated with kernel weight, was subjected to artificial selection during the maize breeding process. *ZmIPT2*-*T* had higher IPT activity than *ZmIPT2*-*C*, and this favorable allele for kernel weight could be used in molecular marker-assisted selection for improvement of grain yield components in Chinese maize breeding programs.

## Background

Genetic improvement of grain yield is important for ensuring food security. Many important agronomic traits, including yield, show continuous phenotypic variation [1]. Grain yield is a complex quantitative trait, and is determined by several components including kernel number and kernel weight [2]. To date, more than 185 quantitative trait loci (QTL) underlying yield components including kernel row number (KRN), kernel number per row (KNPR), and hundred kernel weight (HKW) have been identified across the maize genome using various mapping populations (2012 November update to Gramene database), which has improved our understanding of the genetic basis of maize yield. Therefore, grain size and kernel weight are important targets for artificial selection for high grain yield. For example, QTL associated with *GS3 *[3] and *GW5*/*SW5 *[4,5] have been selected for grain size in rice. Also, QTL associated with *TaGW2*-*6A Hap*-*6A*-*A* for grain size [6] and *TaCKX6*-*D1 *[7] for grain weight have been identified and artificially selected from within Chinese wheat germplasm. Markers derived from QTL controlling similar components of grain yield in maize will be very useful for marker-assisted selection.

Several genes that control kernel weight or kernel number have been cloned in crops, and some of these genes were found to be involved in carbon and nitrogen metabolism [8-10], protein degradation [11,12], and hormone metabolism [13,14]. All of these processes affect the production and export of C- and N-assimilates to the seed, thereby increasing crop yield. C- and N-metabolism are essential to all processes in plants, including reproductive development, but are especially so during grain filling [8]. Among the genes involved in carbon metabolism, rice *grain incomplete filling 1*, which functions similarly to the invertase encoded by *miniature 1* in maize [10], also encodes a cell-wall invertase required for carbon partitioning during early grain-filling [8]. A gene involved in nitrogen metabolism in rice, *OsARG*, encodes an arginine hydrolase that, when overexpressed, increases grain number per plant under nitrogen-limited conditions, due to increased nitrogen remobilization at the reproductive stage [9].

In addition, degradation of proteins via the ubiquitin/proteasome pathway negatively regulates cell division and grain yield. Another gene, *Grain Weight* 2 (*GW2*), which encodes a RING-type E3 ubiquitin ligase, functions in ubiquitin-mediated degradation, and the loss of *GW2* function can enhance rice grain width, weight, and yield [11]. Furthermore, *ZmGW2*-*CHR4*, which functions in the same manner as *GW2*, is also associated with kernel width and kernel weight in maize [12]. Although they have potentially positive impacts on yield, alleles at these loci also should be monitored to avoid possible negative effects of introduced germplasm on yield.

As to the influence of plant hormones, brassinosteroids (BRs) and cytokinins (CKs) are useful for controlling grain yield in crops. BRs stimulate the transport of sucrose and other sugars to the endosperm and embryo. Expression of the maize, rice, or *Arabidopsis thaliana C*-*22* hydroxylase that is involved in BR synthesis in stems, leaves, and roots had clear effects on seed weight [14]. In rice, the accumulation of CK in the inflorescence meristems increases the number of grains per panicle. The rice gene *Gn1a* encodes an oxidase/dehydrogenase (OsCKX2) that functions in the degradation of CKs. An 11-bp deletion in its coding region created a premature stop codon that reduced expression of *OsCKX2* and resulted in increased grain number [13]. In wheat, *TaCKX6*-*D1*, an ortholog of rice *OsCKX2*, was significantly associated with 1000-grain weight by linkage mapping and association analysis [7]. Thus, the CK regulatory pathway likely plays a crucial role in grain yield.

There is also evidence for a role of CKs in balancing the source and sink relationship [15]. Accordingly, exogenous applications of CKs increased seed set and yield stability under heat stress in maize [16]. CKs are co-regulated by isopentenyl transferase (IPT) and oxidase/dehydrogenase in plants. The discovery of biosynthetic enzymes IPT1 through IPT10 [17-19] and degradative enzymes CKX1 through CKX12 [18,20-23] has led to a better understanding of the role of CKs in maize kernel development. Among the *IPTs*, *IPT1* and *IPT10* are highly abundant and are constitutively expressed in all organs, but other IPT transcripts show distinct spatial and temporal patterns of expression [18]. *ZmIPT2* is specifically expressed in the pedicel, endosperm, and embryo [18,19]. *ZmIPT2* consists of an intronless 966 bp coding region for dimethylallyl diphosphate (DMAPP): adenosine triphosphate (ATP)/adenosine diphosphate (ADP) IPT, which preferred ATP and ADP over adenosine monophosphate (AMP) as substrates for IPT activity [19].

To date, the allelic diversity of *ZmIPT2* and the most favorable allele(s) affecting kernel weight have not yet been reported for maize. The objectives of this study were to (1) examine the sequence diversity of *ZmIPT2* in 175 Chinese maize inbred lines; (2) test associations between nucleotide polymorphisms in *ZmIPT2* and various yield components including KRN, KNPR, and HKW, and identify further favorable allele(s) for grain yield components; (3) characterize the IPT activity of the protein products of different alleles *in vitro*; and (4) investigate selection for favorable *ZmIPT2* alleles for kernel weight during maize breeding in China.

## Results

### Phenotypic data

Phenotypic values for individual lines ranged from 7.81 to 18.62 g for KRN (mean = 12.80), from 8.26 to 34.67 g for KNRP (mean = 22.40), and from 12.35 to 47.60 g for HKW (mean = 28.36), as shown in Table 1. KRN was significantly positively correlated with KNPR (*P* <0.01), while HKW was negatively correlated with KNPR (*P* <0.05). Analysis of variance (ANOVA) indicated significant phenotypic variation for KRN, KNPR, and HKW among these 175 maize lines (*P* <0.01). Heritabilities estimated on a per-plot basis were 86.58% for KRN, 84.27% for KNRP, and 86.12% for HKW.

**Table 1:** **Mean squares from ANOVA and correlation coefficients for KRN**, **KNPR**, **and HKW**

**Category**	**Source of variation**	**DF**	**KRN**	**KNPR**	**HKW (g)**
ANOVA	Year	1	0.20	10.3**	2.14
	Replication	2	1.39	0.16	0.44
	Genotype	172	9.81**	10.14**	12.12**
	Year × Genotype	171(169)^a^	1.57**	2.11**	1.95**
Descriptive	Range		7.81-18.62	8.26-34.67	12.35-47.60
Statistics	Mean ± SD		12.80 ± 1.80	22.40 ± 4.55	27.98 ± 5.46
Correlation	KNPR		0.35**		
coefficients	HKW (g)		−0.06	−0.16*	
*h*^*2*^			86.58%	84.27%	86.12%

*DF* degrees of freedom, *HKW* hundred kernel weight, *KRN* kernel row number, *KNPR* kernel number per row, *h*^*2*^ broad-sense heritability; **, *P* <0.01; *, *P* <0.05.

^a^ Because the phenotypic values for some lines were missing for kernel weight, the DF differ. The numbers in parentheses indicate the DF for HKW and the numbers outside parentheses indicate the DF for KRN and KNPR.

### Nucleotide diversity in the *ZmIPT2* coding region

**Figure 1:**
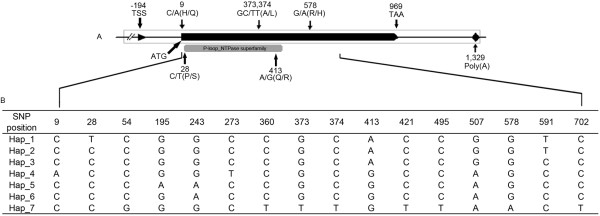
**Nucleotide diversity and amino acid variants in the *ZmIPT2 *coding region. (A)** The full sequence of *ZmIPT2* gene. Sites are labeled according to the position of the corresponding polymorphisms in the aligned sequences, with amino acid alterations indicated in parentheses. The gray box indicates the IPT domain, which starts at position 28 and ends at position 480. **(B)** The *ZmIPT2* genomic sequences grouped into seven haplotypes defined by 16 single nucleotide polymorphisms identified in the alignment.

**Figure 2:**
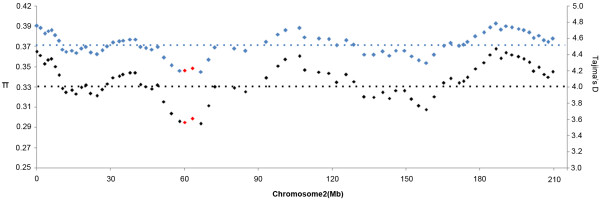
**Sliding-Window analyses of nucleotide diversity *π *and Tajima’s D in the association panel, with a sliding window size of 500 SNP markers and step intervals of 50 SNP markers on maize chromosome 2.** Blue diamonds, *π*; Black diamonds, Tajima’s D; Red diamonds, windows containing the *ZmIPT2* gene locus. Blue dashes represent the mean *π* value; Black dashes represent the mean Tajima’s D value.

**Figure 3:**
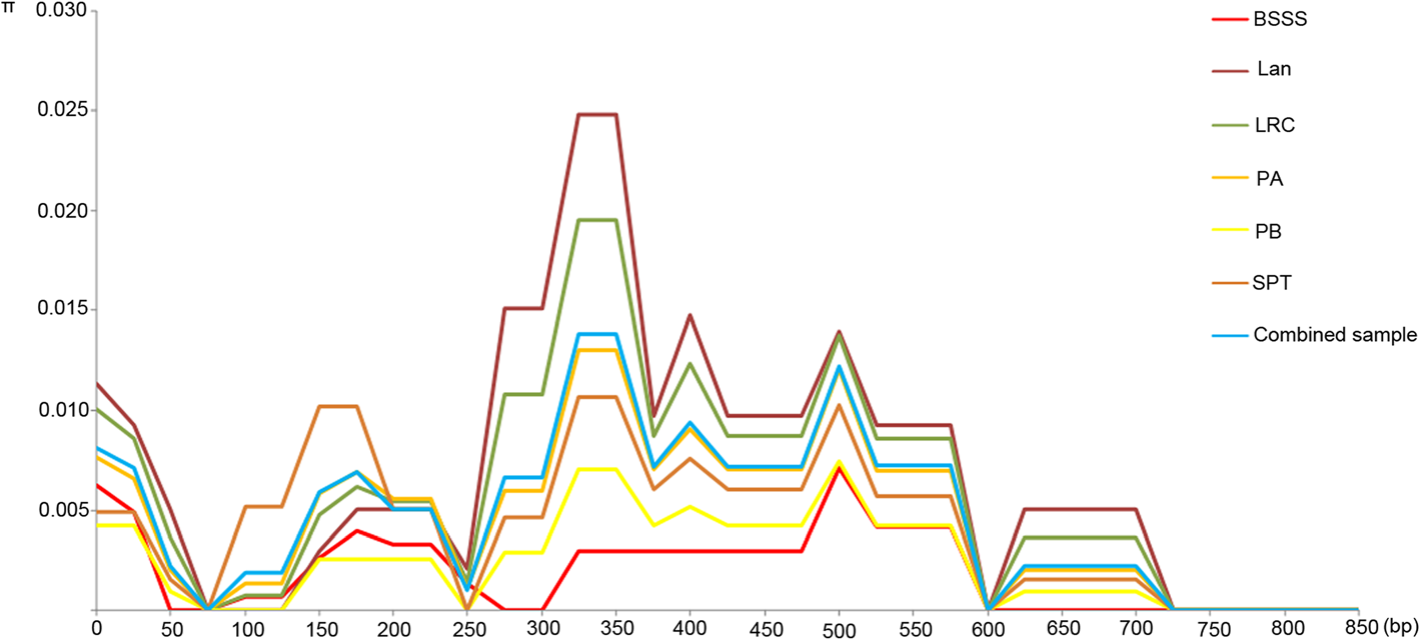
**Sliding-Window analysis of nucleotide diversity in the *ZmIPT2 *coding region across six maize subgroups with 100 bp window and 25 bp step size.** BSSS, derived from US BSSS and Reid germplasm; Lan, derived from US Lancaster Sure Crop germplasm; LRC, derived from Lvda Red Cob, a Chinese landrace; PA, derived from modern US hybrids in China; PB, derived from modern US hybrids in China; SPT, derived from Si-ping-tou, a Chinese landrace. Combined sample, 175 maize inbred lines.

A total of 16 single nucleotide polymorphisms (SNPs) were identified in the *ZmIPT2* coding region, with an average of one SNP every 60 bp, and six of these variants resulted in amino acid substitutions (Figure 1A). Seven haplotypes, named Hap_1 through Hap_7, were detected in our association panel based on these 16 SNPs (Figure 1B). A total of 75 (42.9%) inbred lines harbored Hap_1, while 10, 31, 22, 9, 10, and 18 inbred lines carried Hap_2 through 7, respectively. Hap_1 harbored the *ZmIPT2*-*T* allele at SNP28 (C-T), but other haplotypes bore the *ZmIPT2*-*C* allele. The window covering *ZmIPT2* exhibited relatively lower diversity with a value of 0.347, and it was significantly lower than the average diversity value of 0.372 for Chromosome 2 across 175 maize inbred lines (*P* < 0.01) (Figure 2). Most polymorphisms in *ZmIPT2* were found in the region from 360 to 590 bp, (*P* >0.005) (Figure 3). Among maize subgroups revealed by Xie et al. [24], subgroups Lancaster (Lan) and Lvda red cob (LRC) harbored higher levels of diversity in the *ZmIPT2* coding region, compared with the average for all inbreds (Figure 3).

### Association analysis of KRN, KNPR, and HKW

**Figure 4:**
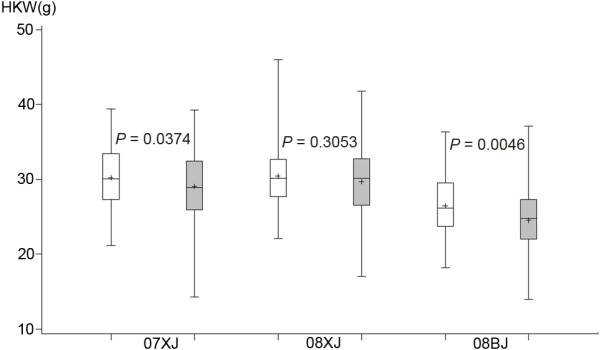
**Hundred-kernel weight (HKW) comparison of groups harboring either *ZmIPT2*-*T *or *ZmIPT2*-*C*.***P*, *P* value for t-test comparing two groups carrying different alleles in each of three environments. 07XJ, Xinjiang in 2007; 08XJ, Xinjiang in 2008; 08BJ, Beijing in 2008. White boxes represent the *ZmIPT2*-*T* group; grey boxes represent the *ZmIPT2*-*C* group.

Association analysis revealed that SNP28 was significantly associated with HKW across all three environments tested (*P* <0.05), and explained 5.42% of the phenotypic variation in HKW at Xinjiang in 2007, 3.41% of the phenotypic variation at Beijing in 2008, and 5.46% of the phenotypic variation at Xinjiang in 2008. Significant association of HKW with SNP195, SNP413, and SNP591 was detected in one or two environments (Table 2). However, only SNP413 was found to be associated with KNPR in Beijing (*P* <0.05), and no SNP was significantly associated with KRN (Table 2). The inbred lines containing the *ZmIPT2*-*T* allele had significantly (*P* <0.05) greater kernel weight than those carrying the *ZmIPT2*-*C* allele, as seen in the data from Xinjiang in 2007 and in Beijing in 2008 across this association panel (Figure 4). The *P* value obtained here supports the strength of the observed association between HKW and SNP28, and improves confidence in identification of the *ZmIPT2*-*T* allele in Hap_1 as the most favorable allele for kernel weight in maize.

**Table 2:** ***ZmIPT2 *polymorphisms associated with KRN**, **KNPR**, **and HKW in the association panel**

**SNPs**	**Genotype**	**Frequency**	**Environment**	**KRN**	**KNPR**	**HKW**
SNP9	C/A	153/22	07XJ	-	-	-
			08BJ	-	-	-
			08XJ	-	-	-
SNP28	T/C	75/100	07XJ	-	-	0.003
			08BJ	-	-	0.0252
			08XJ	-	-	0.0027
SNP54	C/G	157/18	07XJ	-	-	-
			08BJ	-	-	-
			08XJ	-	-	-
SNP195	G/A	166/9	07XJ	-	-	0.0386
			08BJ	-	-	0.0031
			08XJ	-	-	-
SNP243	G/A	156/19	07XJ	-	-	-
			08BJ	-	-	-
			08XJ	-	-	-
SNP413	A/G	115/6	07XJ	-	-	0.0302
			08BJ	-	0.029	-
			08XJ	-	-	0.0351
SNP591	T/C	85/90	07XJ	-	-	-
			08BJ	-	-	-
			08XJ	-	-	0.0187

*HKW* hundred kernel weight, *KRN* kernel row number, *KNPR* kernel number per row, *07XJ* Xinjiang in 2007, *08BJ* Beijing in 2008, *08XJ* Xinjiang in 2008; -, not significant at *P* <0.05.

### Characterization of ZmIPT2

Both the ZmIPT2-T and ZmIPT2-C protein alleles have the same pI and aliphatic index. However, the transition at SNP28 from cytosine (C) to thymine (T) converts proline into serine, resulting in a decrease in the hydropathicity of the entire ZmIPT2 protein from −0.006 to −0.008, as analyzed using GRAVY. The IPT coding region aligned with the P-loop NTPase superfamily that is characteristic of all NTP-binding proteins from position 28 to 480 with an E-value, or probability of a protein sequence of a given bit score occurring by chance, of 1.3e-06 (NCBI blastp database) (Figure 1A). The 3-D structure predicted by SWISS-MODEL (changed to http://swissmodel.expasy.org/) showed that the spatial structure of ZmIPT2-T and ZmIPT2-C differed due to variation in hydrophobicity between the two proteins (Additional file 1). These kinds of changes could affect the binding of substrates to the enzyme, which is mediated by hydrogen bonds [25]. This mutation and resultant differences in physicochemical properties imply that IPT activity could differ between the protein products of the *ZmIPT2*-*T* and *ZmIPT2*-*C* alleles.

**Figure 5:**
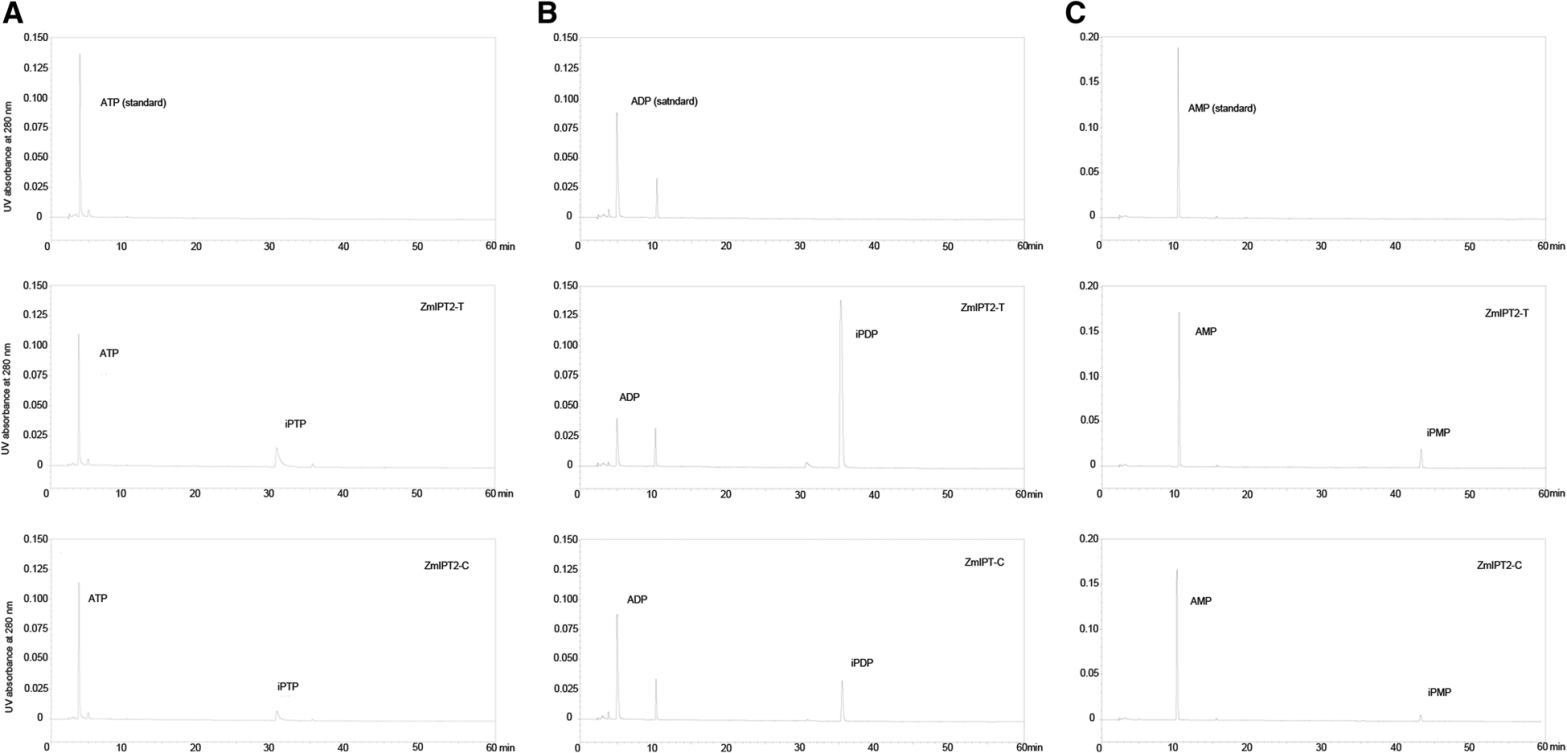
**Determination of isopentenyl transferase activity of ZmIPT2. A**, **B**, and **C** depict determination of DMAPP:ATP, DMAPP:ADP, and DMAPP:AMP isopentenyl transferase activity of recombinant ZmIPT2 protein. iPTP = isopentenyl adenosine triphosphate, iPDP = isopentenyl adenosine diphosphate, and iPMP = isopentenyl adenosine monophosphate.

To further confirm the CK biosynthetic function of these two ZmIPT2 products *in vitro*, *Escherichia coli* prokaryotic expression vectors were constructed to express and purify ZmIPT2 as C-terminal His-tagged recombinant proteins. ZmIPT2-T and ZmIPT2-C purified on Talon® columns reached 95-100% purity, as quantified by Coomassie staining after PAGE (Additional file 2). Purified proteins were then used to assay DMAPP:ATP, DMAPP:ADP, and DMAPP:AMP transferase activity. Figure 5 shows the profile obtained from the reaction mixture of purified ZmIPT2 protein incubated with DMAPP and substrates, including ATP, ADP, and AMP. ZmIPT2-T converted AMP to isopentenyl adenosine monophosphate (iPMP) with higher efficiency than did ZmIPT2-C, as indicated by the amount of iPMP after reaction (Figure 5C). The mean AMP consumption by ZmIPT2-T and ZmIPT2-C were 0.20 μmol and 0.07 μmol, respectively (Additional file 3). Similar results were obtained with ATP or ADP as the substrate (Figure 5A, B). ZmIPT2-T consumed more ADP and ATP, with means of 270.2% and 548.4%, respectively, than consumed by ZmIPT2-C. Additionally, ZmIPT2-T consumed much more ATP and ADP than it did AMP, with means of 7.38- and 13.61-fold the amount of AMP consumed, respectively (Additional file 3). These results indicate that the protein product of the *ZmIPT2*-*T* allele has higher IPT enzyme activity and prefers ADP as substrate, and so could have a more favorable impact on yield.

### Selection in the region of the *ZmIPT2* locus

**Figure 6:**
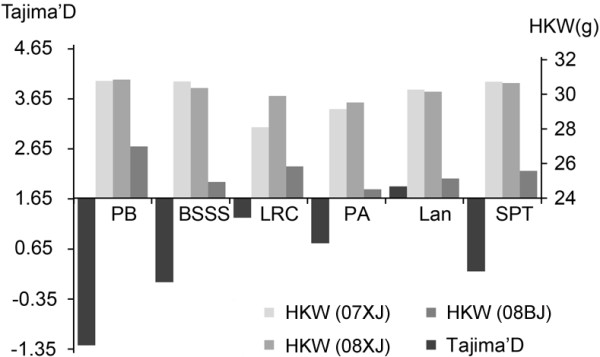
**Tajima’s D values for the *ZmIPT2 *coding region and hundred kernel weights across six maize subgroups.** PB, derived from modern US hybrids in China; BSSS, derived from US BSSS and Reid germplasm; LRC, derived from Lvda Red Cob, a Chinese landrace; PA, derived from modern US hybrids in China; Lan, derived from US Lancaster Sure Crop germplasm; SPT, derived from Si-ping-tou, a Chinese landrace. HKW, hundred-kernel weight. 07XJ, Xinjiang in 2007; 08XJ, Xinjiang in 2008; 08BJ, Beijing in 2008.

A total of 4282 SNPs [26] were used to analyze the effects of selection occurring in this region of Chromosome 2 during selection and breeding. The estimates for π and Tajima’s D in the *ZmIPT2* window showed lower values than the mean estimates for those parameters for all of maize Chromosome 2 (Figure 2). Tajima’s D tests of the *ZmIPT2* coding region and genomic window identified different artificial selection effects across these six maize subgroups, with the largest effect identified in subgroup PB (Figure 6). Notably, 81% of inbred lines in subgroup PB harbored the favorable allele *ZmIPT2*-*T* (Table 3). These results indicate that artificial selection occurred in the genomic region harboring *ZmIPT2* during maize breeding.

**Table 3:** **Distribution of the *ZmIPT2***-***T *allele among six subgroups in 175 Chinese inbred lines**

	**PB**	**BSSS**	**LRC**	**PA**	**Lan**	**SPT**
*ZmIPT2*-*T*	17	18	11	19	5	5
No.^a^	21	29	27	55	18	25
Frequency^b^	81%	62%	41%	35%	28%	20%

^a^ The number of inbred lines in each subgroup.

^b^ The frequency of the *ZmIPT2*-*T* allele in each subgroup.

## Discussion

### Grain yield is a complex trait controlled by minor genes

Grain yield is one of the most important and complex traits in maize. Although many studies on the genetic basis of maize yield-related traits have been reported, few favorable alleles for yield candidate genes have been identified because single genes identified in association studies explain only a small proportion of phenotypic variation. In this study, SNP28 in the *ZmIPT2* coding region was significantly associated with kernel weight across three environments, explaining 4.76% of phenotypic variation. Relatively small allelic effects were also found for one polymorphism in the *ZmGS3* region, which explained 6.29% of variation in Beijing and 7.73% of that in Hainan [27], and another SNP, S40 in the *ZmGW2*-*CHR4* region, which accounted for less than 10% of variation across three environments [12]. Thus, the identification and pyramiding of a large number of favorable alleles will be the key to molecular marker-assisted breeding of high-yielding maize.

### Association mapping of complex traits

As an effective way to dissect complex traits and identify favorable alleles or haplotypes, association mapping has been widely used for many maize traits, including flowering time [28], leaf architecture [29], plant height [26], and disease resistance [30-32]. With candidate-gene association mapping, excellent allelic variants of many genes have been identified, such as *crtRB1* for β-carotenoid synthesis in maize kernels [33]; *Dwarf8* for maize flowering time [6,34,35]; *ZmGW2*-*CHR4*[36] and *ZmGS3*[27], which are related to maize kernel size and weight; *TaGW2*, which is associated with wheat kernel weight and maturity [37]; and other genes related to starch synthesis in rice [38].

### Contribution of CKs to grain yield

Plant hormones control many aspects of development, and like other hormones, CKs affect many plant physiological and biochemical processes, including those controlling delayed senescence [39], suppression of auxin-induced apical dominance [40], signaling of nitrogen availability [41], cell division and differentiation [42], and sink strength [15]. IPT proteins, which are involved in synthesis of CKs, are encoded by a multi-gene family in maize [17-19], rice [43], soybean [44], *Arabidopsis *[45,46], petunia [47], hop [48] and pea [49]. A favorable IPT allele that resulted in increased grain weight has now been identified in maize using an association mapping approach (Figure 3).

CK content is co-regulated by two families of genes, including isopentenyl transferase and an oxidase/dehydrogenase in plants. In rice, reduced expression of *OsCKX2*, which encodes the oxidase/dehydrogenase, caused an increase in grain number per panicle, due to CK accumulation in inflorescence meristems. *OsCKX2* is expressed strongly in pistils, at an intermediate level in inflorescences and seeds, and at low levels in other organs [13]. *TaCKX6*-*D1*, a wheat ortholog of rice *OsCKX2*, was related to HGW by linkage mapping and association analysis. *TaCKX6*_*D1* was expressed at a high level in seeds and pistils, but at a low level in other organs [7]. One study showed that *ZmIPT2* is specifically expressed in pedicel, endosperm, and embryo, and plays a major role in CK biosynthesis for maize kernel development [18,19]. In this study, however, only SNP413 was found to be significantly associated with KNPR in one of the three experimental environments (Beijing), and no associated SNP was found for KRN (Table 2). CKs coordinately induce extracellular invertase and hexose transporter activity, which are functionally coupled to supply carbohydrates to sink tissues [15]. In this study, a single nucleotide substitution from C to T in the *ZmIPT2* coding region was significantly associated with kernel weight. This suggests that manipulation of CK metabolism in reproductive organs could be an effective approach to further increase crop grain yields, by increasing the flow of assimilates from source to sink, or by increasing sink capacity.

*In vitro* characterization of the ZmIPT2 enzyme showed that it prefers ADP and ATP over AMP as substrates for DMAPP IPT activity (Figure 5, Additional file 3), consistent with the results of a previous study [19]. Six amino acid substitutions, none of which were conservative, occurred in ZmIPT2, compared with 10 other maize ZmIPTs [18], indicating that ZmIPT2, and its specific expression pattern, is conserved and essential for kernel development. Interestingly, the mutation associated with the favorable allele, *ZmIPT2*-*T*, was detected in the *ZmIPT2* coding region, and found to result in a serine to proline substitution in the conserved IPT domain that caused the ZmIPT2-T enzyme to consume more ATP, ADP, and AMP than the ZmIPT2-C enzyme (Figure 5, Additional file 3). Thus, selection for the molecular marker SNP28 should improve yield by positively influencing maize kernel development via an increase in IPT enzyme activity.

### Nucleotide diversity in *ZmIPT2* across six subgroups

In this study, the coding region of *ZmIPT2* was sequenced across 175 Chinese maize inbreds. The coding regions of functional genes tend to be relatively conserved, due to their specificity for and affinity with other types of molecules. Thus, under natural selection, beneficial variants tend to become gradually fixed, while detrimental variants tend to be eliminated [37]. Lower levels of diversity within the *ZmIPT2* locus were estimated in subgroups PB and BSSS, compared to the combined group (*P* < 0.01) (Figure 3), suggesting either that the germplasm base remained remarkably narrow or that strong positive selection at that locus occurred in these subgroups. According to pedigrees, 17 inbreds in subgroup PB were mainly derived from US hybrid P78599. However, subgroup Lan harbored the most diversity in the *ZmIPT2* coding region across all these inbreds (Table 1; Figure 3). This subgroup has been identified as having the most genetic diversity in one study using 145 SSR loci randomly distributed across the genome [50].

### Selection for grain yield during the breeding process

It is hypothesized that natural selection favored smaller-seeded wild ancestors with a larger number of seeds per plant, earlier maturity, and wider geographic distribution [51]. However, high crop yield associated with larger grain size and weight has been the objective of artificial selection during most breeding programs. For example, the genes *GS3 *[52] and *GW5*/*SW5 *[3,53] have been proven to be involved in the evolution of grain size in rice. Unlike rice, in wheat and maize, artificial selection is associated with an almost uniform increase in seed or grain size. The allele *TaGW2*-*6A Hap*-*6A*-*A*, which was associated with larger grain size, increased in frequency under positive selection from 50.0% in the 1950s to current levels of 77.42% [37]. Under strong selection at a given locus, domestication has the potential to drastically increase LD and reduce diversity [4].

Estimates of the parameters π and Tajima’s D in this study showed that artificial selection took place at the *ZmIPT2* locus (Figure 3). However, only Hap_1, among seven haplotypes examined here, harbored the *ZmIPT2*-*T* allele (Figure 1), and this favorable allele was associated with increased grain weight in two of the three experimental environments (Figure 4), suggesting positive selection for higher grain weight. Similarly, a 95.7% reduction in nucleotide diversity across the *GS3* locus occurred among rice accessions carrying the A allele for *GS3 *[52]. The current study found that the *ZmIPT2*-*T* allele was not evenly distributed among these six maize subgroups, occurring with the highest frequency in subgroup PB, in which 81% of the tested lines harbor the favorable allele (Table 3). The lowest Tajima’s D value was also found for subgroup PB (Figure 6) indicating that distinct positive selection pressure has taken place at the *ZmIPT2* locus in this subgroup. Selection for the favorable allele *ZmIPT2*-*T* within each subgroup should be continued in order to improve maize yield.

## Conclusions

A favorable allele, *ZmIPT2*-*T*, was associated with kernel weight in Chinese maize germplasm. ZmIPT2-T was shown *in vitro* to have higher IPT activity than ZmIPT2-C with ADP, ATP, or AMP as substrate. Artificial selection at the *ZmIPT2* locus was detected with Tajima’s D tests across six subgroups of Chinese maize germplasm, with the most frequent favorable allele identified in subgroup PB. This favorable allele could be used in molecular marker-assisted selection for improvement of grain yield components in Chinese maize breeding programs.

## Methods

### Experimental design and statistical analyses

In this study, a total of 175 maize inbred lines were used, which were subdivided into six subgroups [24], including 29 BSSS lines derived from US BSSS and Reid germplasm; 55 PA (Partner A) lines derived from modern US hybrids in China; 21 PB (Partner B, harboring the distinct heterosis reaction of PA) lines derived from modern US hybrids in China; 18 Lan lines derived from US Lancaster Sure Crop germplasm; 27 LRC lines derived from Lvda Red Cob, a Chinese landrace; and 25 SPT lines derived from Si-ping-tou, a Chinese landrace. All lines were planted at Xinjiang in 2007 and 2008, and at Beijing in 2008 to measure grain yield components, including KRN, KNPR, and HKW. A randomized complete-block design was employed with three replications of 20 plants of each line per location planted in 4.5-m rows, 0.6 m apart. Normal maize agricultural practices were carried out. At maturity, all ears were harvested manually and dried to grain moisture of 13%. KRN was scored as number of rows per ear, KNPR was scored as total kernels in a row per ear, and HKW was measured on 100 randomly selected kernels per ear. The average phenotypic values of each plot over three replications in each environment were used for final analysis.

Descriptive statistics and analysis of variance (ANOVA) for KRN, KNPR, and HKW were obtained using the program PROC GLM in SAS software version 9.13 (Copyright © 2009 SAS Institute Inc., 2009. SAS and all other SAS Institute Inc. product or service names are registered trademarks or trademarks of SAS Institute Inc., Cary, NC, USA). The SAS program PROC CORR was used to quantify the relationship among the three traits. Broad-sense heritability was estimated based on plot as *h*^*2*^ = σ_*g*_^2^/ (σ_*g*_^2^ + σ_*gl*_^2^/*n* + σ_*e*_^2^/*nr*), where σ_*g*_^*2 *^is genetic variance, σ_*gl*_^*2*^ is genotype-by-environment interaction, *σ*_*e*_^*2*^ is error variance, *r* is the number of replications, and *n* is the number of locations. The estimates for *σ*_*g*_^*2*^, *σ*_*gl*_^*2*^, and *σ*_*e*_^*2*^ were acquired from ANOVA [5].

### DNA isolation, PCR amplification and DNA sequencing

Genomic DNA was extracted from maize leaves using the CTAB method [54]. Primers ZmIPT2-5′ (5′-ATCATCAAGACAATGGAGCACGGTG-3′) and ZmIPT2-3′ (5′-CGTCCGCTAGCTACTTATGCATCAG-3′) were designed based on the published ZmIPT2 cDNA sequence (Accession number EU263126). *ZmIPT2* genomic sequences were amplified from each of 175 inbred lines in 25 *μ*L reaction mixtures containing 20 ng of genomic DNA, 0.2 mM of each dNTP, 1 *μ*M of each primer, 2 × GC buffer (2 mM Mg^2+^), and 2.5 U TransTaq High Fidelity DNA polymerase (Transgen Biotechnology Corporation, Beijing, China). Touchdown PCR was applied as follows: 94°C for 2 min (one cycle);94°C for 30 s, 65°C for 45 s, and 72°C for 90 s (5 cycles, annealing temperature reduced 1°C per cycle);94°C for 30 s, 60°C for 45 s and 72°C for 1 min 30 s (30 cycles); 72°C for 7 min, then the reaction was then held at 4°C. The products were sequenced (ABI3730) at the public laboratory of the National Key Facility of Crop Gene Resources and Genetic Improvement, Institute of Crop Science, Chinese Academy of Agricultural Science.

### Nucleotide diversity and selection in breeding process

DNA sequences of all lines were analyzed using DnaSP Version 4.00 [55]. The Sliding-Window analysis of nucleotide diversity was performed with window size set at 100 bp and steps of 25 bp. The parameter *π* was estimated as the average proportion of nucleotide differences between all possible pairs of sequences in the sample [56]. A total of 4282 SNPs on maize Chromosome 2 from the MaizeSNP50 BeadChip (Illumina, Inc.) were used to estimate *π* and Tajima’s D using TASSEL 3.0 software [26,57].

### Association mapping of KRN, KNPR and HKW

To exclude the effect of population structure on association mapping, population structure and kinship information for 175 lines were analyzed using STRUCTURE version 2.3 and SPAGeDi software with SNPs selected from a collection of 5000 SNPs identified in a previous study and selected based on physical position and minor allele frequency [26]. Association mapping between three yield-related traits and the nucleotide diversity of the *ZmIPT2* coding region was performed using TASSEL 3.0 [57] with a mixed linear model (MLM) controlling both population structure (Q) and relative kinship (K).

### Characterization of ZmIPT2

The 3D protein model of ZmIPT2 was predicted using the online process SWISS-MODEL Automated Mode (http://swissmodel.expasy.org/). Physicochemical properties of ZmIPT2 proteins, including isoelectric point (pI), aliphatic index, and grand average of hydropathicity (GRAVY) were analyzed using ProtParam (http://web.expasy.org/protparam/). These kinds of changes could affect the binding of substrates to the enzyme, which is mediated by hydrogen bonds [25]. The conserved domain was used to query the smart web program (http://smart.embl-heidelberg.de/).

To characterize the enzyme activity of the products of these two *ZmIPT2* alleles (the T variant and the C variant), prokaryotic expression and enzyme activity determination of ZmIPT2 were carried out *in vitro*. The gene fragments harboring *ZmIPT2*-*T* or *ZmIPT2*-*C* were amplified using gene-specific primers with appropriate *Nde*I and *Not*I restriction site extensions, (5′-GGCATATGGAGCACGGTGCCGTCGC-3′ and 5′-CCGCGGCCGCTCATCATGCATCAGCCACGGCGGTGA-3′) from the ‘Ye478’ (Hap_1) and ‘Guan17’ (Hap_2) inbred lines, respectively. The PCR products were digested with *Nde*I and *Not*I, and then cloned into pET30b (Novagen, Darmstadt, Germany) at these same restriction enzyme sites. The recombinant plasmids were integrated into BL21 (DE3) *E*. *coli* competent cells (Tiangen Biotech Co., Ltd, Beijing, China). The cultures were incubated at 37°C with shaking until log phase at OD_600_ = ~ 0.4. Expression of the IPT protein was induced by incubation with 1 mM IPTG at 37°C for 3 h, followed by purification using the HisTALON™ Gravity column Purification Kit (Clontech Laboratories, Inc., CN) according to the manufacturer’s guidelines. Sodium dodecyl sulfate (SDS)-polyacrylamide gel electrophoresis analysis (PAGE) was performed to ensure purity, and protein density was measured using the Macro-BCA Assay Kit (Gragen Life Science Inc., CN).

Purified protein was used to determine DMAPP:ATP, DMAPP:ADP, and DMAPP:AMP IPT activities. Each purified protein (~0.13 mg/ml) was incubated in a reaction mixture containing 12.5 mM Tris–HCl (pH 7.5), 37.5 mM KCl, 5 mM MgCl_2_, 1 mM DMAPP (Sigma-Aldrich Co., St. Louis, USA), and 1 mM ATP (Sigma-Aldrich Co., St. Louis, USA), ADP (Sigma-Aldrich Co., St. Louis, USA) or AMP (Sigma-Aldrich Co., St. Louis, USA) for 2 h at 30°C. The reaction was stopped by boiling for 5 min. The reaction products were separated by reversed-phase HPLC (Shimadzu LC-10AT series HPLC system with a SPD-10AVP UV–vis detector) with a C_18_-ODS_2_ column (Kromasil), according to the following program: 20 mM KH_2_PO_4_ for 15 min, linear gradient of 0% acetonitrile, 20 mM KH_2_PO_4_ to 20% acetonitrile, and 4 mM KH_2_PO_4_ over 30 min. UV absorbance was monitored at 280 nm.

## Abbreviations

ANOVA: Analysis of variance; BRs: Brassinosteroids; CKs: Cytokinins; DMAPP: Dimethylallyl diphospate; GLM: General linear model; HKW: Hundred kernel weight; IPT: Isopentenyl transferase; KRN: Kernel row number; KNPR: Kernel number per row; LD: Linkage disequilibrium; MLM: Mixed linear model; QTL: Quantitative trait loci; SNP: Single nucleotide polymorphisms; 07XJ: Xinjiang location in 2007; 08BJ: Beijing location in 2008; 08XJ: Xinjiang location in 2008.

## Competing interests

The authors declare that they have no competing interests.

## Authors’ contributions

SZ and XL conceived and designed the experiments. JW and BL performed the experiments. JW, BL, CL, XY, HW, ZH, ML, DZ, and XC contributed reagents, materials, and analysis tools. JW, BL, and XL wrote the manuscript. SZ, XL, and JW coordinated the research. All authors read and approved the final manuscript.

## Supplementary Material

Additional file 1 **3-D structure of ZmIPT2 predicted with the program Swiss-PdbViewer.** Ser and Pro represent amino acids in ZmIPT2-T and ZmIPT2-C, respectively. Click here for file

Additional file 2 Purified recombinant proteins for enzyme activity determination. Click here for file

Additional file 3 **The consumption of ATP, ADP, and AMP by ZmIPT2-C and ZmIPT2-T during *in vitro *enzyme activity determination.** Click here for file
