# Supplementary figures and images for: A non-synonymous SNP within the isopentenyl transferase 2 locus is associated with kernel weight in Chinese maize inbreds (Zea mays L.)
Source: BMC Plant Biol. 2013 Jul 5;13:98. doi: 10.1186/1471-2229-13-98 (PMC3704264; doi:10.1186/1471-2229-13-98)

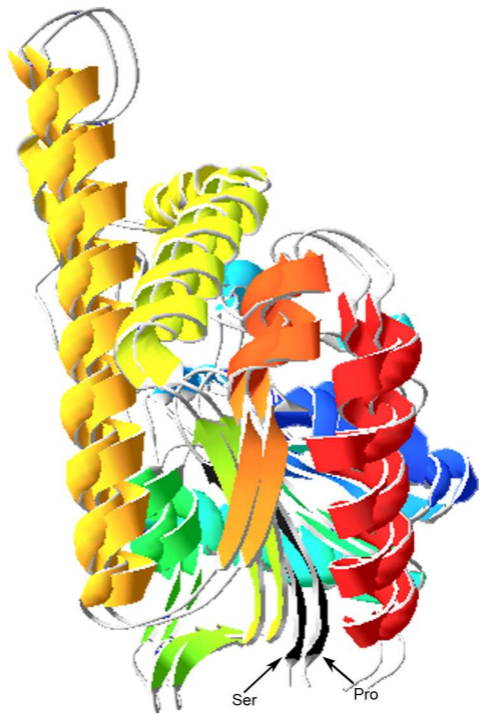

Supplement: Additional file 1 — 3-D structure of ZmIPT2 predicted with the program Swiss-PdbViewer. Ser and Pro represent amino acids in ZmIPT2-T and ZmIPT2-C, respectively. [file 1471-2229-13-98-S1.pdf]

kDa

MM

ZmIPT2-T

ZmIPT2-C

250

130

95

72

55

36

28

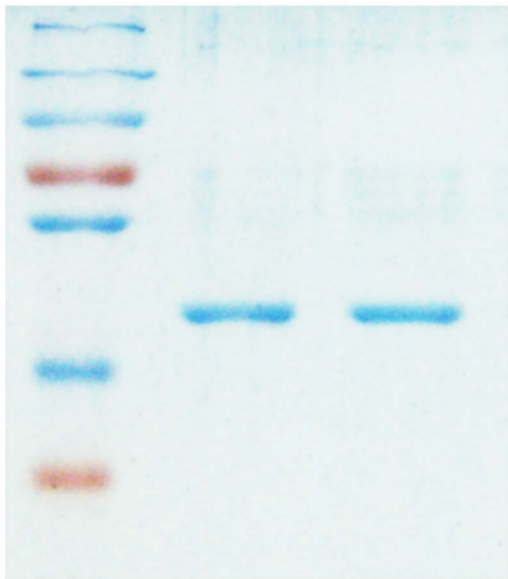

Supplement: Additional file 2 — Purified recombinant proteins for enzyme activity determination. [file 1471-2229-13-98-S2.pdf]

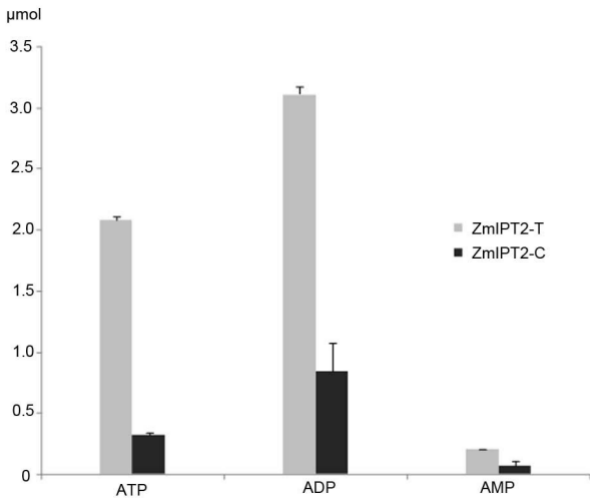

Supplement: Additional file 3 — The consumption of ATP, ADP, and AMP by ZmIPT2-C and ZmIPT2-T during in vitro enzyme activity determination. [file 1471-2229-13-98-S3.pdf]
